# Advances in Medicalized Hair Loss Solutions: A Review of Current Clinical Practices and Regenerative Medicine-Based Protocols with Focus on Off-Label Injectable Treatments

**DOI:** 10.3390/jcm15051836

**Published:** 2026-02-27

**Authors:** Angelica Ferro, Mohammad Alkhowailed, Alexandre Porcello, Marco Cerrano, Michèle Chemali, Kelly Lourenço, Cíntia Marques, Wassim Raffoul, Lee Ann Applegate, Alexis E. Laurent

**Affiliations:** 1 Development Department, LOUNA REGENERATIVE SA, CH-1207 Geneva, Switzerland; a.ferro@louna-aesthetics.com (A.F.); a.porcello@louna-aesthetics.com (A.P.); k.lourenco@louna-aesthetics.com (K.L.); c.marques@louna-aesthetics.com (C.M.); 2 Department of Dermatology, College of Medicine, Qassim University, Buraidah 52571, Saudi Arabia; m.alkhowailed@qu.edu.sa; 3 Aesthetic Surgery Department, Entourage Aesthetic Clinic, CH-1003 Lausanne, Switzerland; m.cerrano@entourage.ch; 4 Plastic and Aesthetic Surgery Service, Centre Médical Lausanne Ouest, CH-1008 Prilly, Switzerland; m.chemali@cmlo.ch; 5 Plastic and Reconstructive Surgery, Ensemble Hospitalier de la Côte, CH-1110 Morges, Switzerland; wassim.raffoul@ehc.vd.ch; 6 Faculty of Biology and Medicine, University of Lausanne, CH-1015 Lausanne, Switzerland; lee.laurent-applegate@unil.ch; 7 Center for Applied Biotechnology and Molecular Medicine, University of Zurich, CH-8057 Zurich, Switzerland; 8 Oxford OSCAR Suzhou Center, Oxford University, Suzhou 215123, China; 9 Manufacturing Department, LAM Biotechnologies SA, CH-1066 Epalinges, Switzerland; 10 Manufacturing Department, TEC-PHARMA SA, CH-1038 Bercher, Switzerland

**Keywords:** alopecia, clinical studies, hair follicle reactivation, hair loss, injectable treatments, off-label treatments, regenerative medicine, hair regrowth, treatment protocols

## Abstract

Hair loss, or alopecia, constitutes a significant and prevalent concern affecting individuals worldwide. Despite the availability of numerous commercial solutions, many individuals continue to experience substantial psychological distress, leading to adverse impact on personal relationships, social interactions, and occupational performance. The limitations of conventional treatments, such as oral medication with potential systemic side effects and topical applications with localized adverse events, have driven the exploration of alternative therapies. Emerging localized injectable treatments for hair regrowth (PRP, stem cells, exosomes) offer a promising avenue for addressing this persistent issue. These injectable therapies hold the potential to minimize the systemic side effects often associated with oral medications, while also mitigating the localized adverse events that can arise from topical applications. This narrative review provides a comprehensive overview of the medical state-of-the-art in off-label injectable hair regrowth treatments, delving into the diverse range of available options. A critical component of this narrative review involves a thorough evaluation of relevant clinical studies, assessing the efficacy and safety profiles of these emerging therapies. Furthermore, detailed attention is given to injection techniques and administration protocols, crucial factors in optimizing treatment outcomes. These evolving therapies represent a significant advancement in the field of scalp regenerative medicine. By stimulating hair follicle reactivation, these treatments aim to promote sustained and natural hair growth, providing individuals with more effective and durable solutions. The enhanced safety profiles of these injectable therapies, compared to conventional systemic pharmacological treatments (minoxidil, finasteride), offer a substantial improvement in patient care, addressing a widespread clinical need.

## 1. Introduction

Hair loss, or alopecia, is a complex and multifaceted condition that significantly impacts the psychological and social well-being of affected individuals. It is broadly classified into two primary categories: noncicatricial (non-scarring) and cicatricial (scarring) alopecia [1,2,3,4]. Noncicatricial alopecia, characterized by the preservation of hair follicle integrity, offers the potential for hair regrowth, contrasting sharply with cicatricial alopecia, where follicular destruction leads to irreversible hair loss [4,5].

Within the realm of noncicatricial alopecia, androgenetic alopecia (AGA), commonly known as male pattern hair loss (MPHL) or female pattern hair loss (FPHL), remains the most prevalent form, accounting for 80% of men and 50% of women at the age of 70 years old [4,6,7]. AGA is most seen in Caucasians, followed by Asians and African Americans, and finally Native Americans and Eskimos [6,7]. It arises after puberty, and is characterized by gradual hair thinning that affects the crown and frontal areas of the scalp in men, the frontal and vertex scalp in women [6,7,8]. Moreover, hairline recession often occurs in men, while the frontal hairline is usually spared in women [6,7]. Diffuse hair loss uniformly affects the scalp, with telogen effluvium (TE) being the most frequent type, causing the loss of over 200 hairs daily [4]. Alopecia areata (AA), an autoimmune disorder that can affect individuals of any age, sex, race, or ethnicity, is the most common type of focal hair loss, often developing in childhood [4].

The hair follicle cycle undergoes life-long transformations into four different phases: anagen, growth; catagen, regression; telogen, rest; and exogen, shedding [9,10,11,12]. Alopecia alters the balance or timing of these steps, causing premature transitions between phases. Thus, hair may stop growing, enter the resting phase early, or shed excessively, ultimately leading to noticeable hair thinning or loss [12,13]. Furthermore, hair health is influenced by many variables such as genetic factors or predisposition, stress and depression, infections, medication, chemical exposure, lifestyle habits, childbirth, and hormones [12].

For instance, although AGA is a result of the overstimulation of androgen receptors (AR), genetics significantly influence the outcome [13]. Abnormalities in AR and 5-α reductase genes have been associated with AGA. In addition, the AR gene on the X chromosome is linked to the ectodysplasin A2 receptor (EDAR2) gene, with mutations in this gene being observed in AGA [13]. AGA is also related to genetic alteration of the Wnt signaling pathway, affecting dermal papilla cell proliferation and androgen metabolism [13]. Recent research continues to unravel the intricate genetic and hormonal underpinnings of AGA, highlighting the pivotal role of genetic predispositions and the androgen signaling pathway in its pathogenesis [14]. Notably, advancements in genetic sequencing and analysis have facilitated the identification of specific gene variants associated with AGA, providing deeper insights into the heritability and molecular mechanisms of this condition. While research continues to further define genetic causes, it is clear that multiple genetic factors contribute to the condition.

Diffuse hair loss, exemplified by TE, and focal hair loss, most notably AA, present distinct clinical challenges. TE is often triggered by physiological stressors, hormonal fluctuations, nutritional deficiencies, or certain medications, leading to a temporary but significant increase in hair shedding. Conversely, AA, an autoimmune disorder, is characterized by the immune system’s attack on hair follicles, resulting in localized hair loss. Recent advancements in understanding the immunopathogenesis of AA have led to the development of targeted therapies, particularly Janus kinase (JAK) inhibitors, which have revolutionized the treatment landscape [15]. The development and approval of JAK inhibitors have marked a significant milestone in the treatment of AA. These drugs target the inflammatory pathways involved in AA by inhibiting JAK enzymes, thereby modulating cytokine signaling and reducing immune-mediated follicular damage [15]. Clinical trials have demonstrated the efficacy and safety of JAK inhibitors in promoting substantial hair regrowth in patients with severe AA, offering hope to those who previously had limited treatment options [15]. This advancement represents a paradigm shift in AA management, providing a targeted and effective therapeutic approach.

To date, there are several available therapies for AGA, such as lifestyle changes, hair care routines, and medicines. However, only two medications, topical minoxidil (i.e., for both men and women) and oral finasteride (for men), are approved by the US Food and Drug Administration (FDA) for this condition [6,12,13]. Originally introduced in the 1970s as an antihypertensive, minoxidil is the first FDA-approved drug for AGA and is also used off-label for other hair loss conditions. It is metabolized into its active form, minoxidil sulfate, which acts as a vasodilator by activating potassium channels in peripheral artery smooth muscle, promoting cell proliferation. Additionally, minoxidil boosts vascular endothelial growth factor (VEGF) in dermal papilla cells and stimulates prostaglandin E_2_ production, prolonging the anagen phase [6,12]. Finasteride has been approved for the treatment of MPHL since 1997. It works by inhibiting 5-α-reductase type 2, which prevents the conversion of testosterone to dihydrotestosterone (DHT) and helps reduce androgen-driven follicular miniaturization [6,13]. Despite being effective for hair loss, both minoxidil and finasteride have notable drawbacks, and stopping treatment can rapidly accelerate hair loss, resulting in a decline in their prescription and the search for alternative therapeutic approaches [13].

Importantly, the hair follicle cycle is a crucial target for hair loss therapies. Disruption in this cycle, such as premature entry into the catagen or telogen phases, contributes to various forms of alopecia. Research continues to investigate the complex molecular mechanisms that regulate these phases, aiming to identify novel therapeutic targets and strategies for modulating hair follicle cycling. Advances in stem cell biology, hair transplantation, and regenerative medicine have also opened new avenues for hair follicle regeneration and restoration. Current research is actively exploring novel drug delivery methods to enhance the efficacy and safety of hair loss treatments. For instance, microneedle patches are being investigated for their ability to deliver therapeutics directly to hair follicles, bypassing the stratum corneum and potentially improving drug penetration and bioavailability [16]. This method offers the advantage of localized drug delivery, minimizing systemic exposure and reducing the risk of systemic side effects. Additionally, microneedle patches offer a minimally invasive and patient-friendly alternative to traditional injection methods, potentially improving patient compliance and treatment outcomes. Another technique that has been explored for the management of AGA and AA is carboxytherapy [17,18]. This procedure involves the intradermal insufflation of sterile, medical grade carbon dioxide (CO_2_) [17]. In one clinical trial evaluating its use in both conditions, carboxytherapy was associated with statistically significant improvements in clinical scores, global assessments, and dermoscopic and digital trichoscopic parameters compared with the placebo [19]. Additional studies have reported that combining carboxytherapy with intralesional corticosteroids or topical minoxidil may lead to further improvement [20,21]. 

This narrative review focuses on the medical state-of-the-art in off-label injectable treatments for hair loss, examining their clinical applications, effectiveness, and safety profiles. Importantly, very few injectable solutions are approved for this indication, which explains the widespread clinical practices of such off-label treatment. Based on this real-world clinical practice fact, this review was designed to cover the effectively used preparations and protocols. Although this review is narrative in design, a structured search strategy was applied to enhance transparency. Publications were identified via PubMed/MEDLINE, supplemented by Scopus and Google Scholar, covering January 2000 to December 2025, with additional screening of www.ClinicalTrials.gov for registered or ongoing trials. Search strings combined alopecia entities (e.g., “androgenetic alopecia”, “female pattern hair loss”, “alopecia areata”, “telogen effluvium”, “hair loss”) with intervention terms relevant to injectable or injection-adjacent practice (e.g., “platelet-rich plasma/PRP”, “stem cell/ADSC/SVF/micrograft”, “conditioned media”, “extracellular vesicle” OR “exosome”, “botulinum toxin”, “hyaluronic acid”, “polynucleotide” OR “polydeoxyribonucleotide/PDRN”, “hair booster”, “mesotherapy”, “intradermal”, “subcutaneous”, “scalp injection”), and when needed to capture mechanistic papers not indexed under hair loss terms (e.g., “dermal papilla”, “hair follicle stem cell”, “anagen”, “Wnt/β-catenin”, “angiogenesis”, “inflammation/oxidative stress”). Reference lists of included articles were hand-searched. Eligible records comprised clinical studies of scalp injection/mesotherapy approaches and relevant systematic reviews, complemented by selected translational studies informing plausibility and safety. No formal risk-of-bias tool was applied; studies were qualitatively appraised based on design, outcome objectivity, and safety reporting. Grey literature (e.g., product dossiers/technical documents) was included to reflect real-world practice and was explicitly labeled as non-peer reviewed. Therefore, this review will provide a comprehensive summary of current clinical studies, injection techniques, and potential benefits and risks associated with injectable therapies. By synthesizing the latest research and clinical practices, this review aims to contribute to the understanding of injectable therapies in hair restoration and to inspire future research in this dynamic and rapidly evolving field. Moreover, we hope to provide a comprehensive overview of the current landscape of research to help medical professionals and researchers alike.

## 2. Off-Label Injectable Treatments Clinically Used to Mitigate Hair Loss

**Figure 1:**
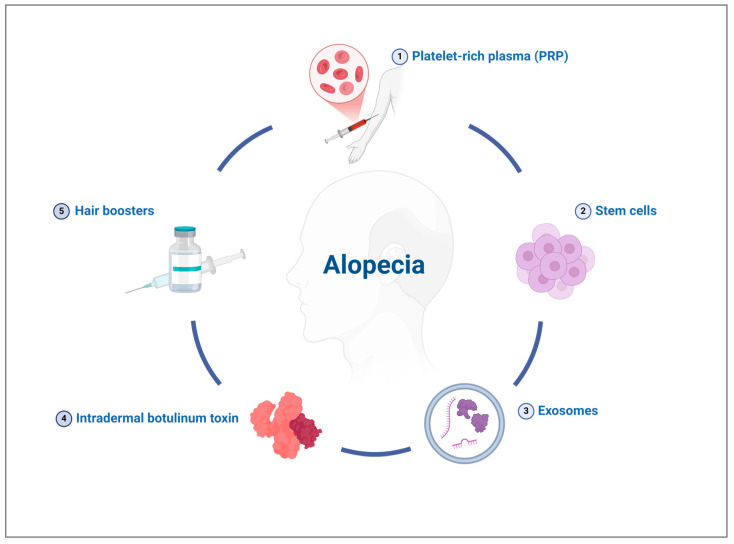
Overview of commercialized products and protocol categories which are currently used as off-label injectable treatments for alopecia.

Despite increasing efforts to develop effective solutions against hair loss, only two drugs have received FDA approval for treating alopecia. Parallelly, several off-label treatments have emerged in clinical use and have demonstrated effectiveness in managing AGA in various studies (Figure 1) [5].

Some of these therapies are relatively recent and still require further clinical investigation, highlighting the importance of an updated literature review. The following paragraphs thus describe the medical state-of-the-art in off-label injectable hair regrowth treatments based on available clinical reports.

### 2.1. Use of Platelet-Rich Plasma (PRP) for Hair Loss Management

PRP represents a concentrated biological product derived from autologous blood, enriched with platelets, fibrinogen, fibrin, chemokines, and leukocytes [22,23,24,25,26,27,28]. This complex mixture has gained significant traction across various medical disciplines, including oral and maxillofacial surgery, orthopedics, wound healing, and, increasingly, medical and cosmetic dermatology [24,25,26,27,28,29]. The clinical efficacy of PRP is intrinsically linked to the concentration of platelets and the subsequent release of a plethora of growth factors, notably VEGF, platelet-derived growth factor (PDGF), transforming growth factor beta (TGF-β1 and TGF-β2), epithelial growth factor (EGF), and insulin-like growth factor (IGF) [27,28]. Upon activation, PRP orchestrates a cascade of biological events, releasing these potent growth factors that can effectively transition telogen hair follicles back into the anagen growth phase. These growth factors exert pleiotropic effects, stimulating mitogenesis and differentiation of crucial cellular components, including stem cells, fibroblasts, keratinocytes, and endothelial cells. Furthermore, they play a pivotal role in the healing process by promoting angiogenesis, cellular proliferation, differentiation, chemotaxis, and tissue morphogenesis [23,25,26,28]. Consequently, PRP has emerged as a promising therapeutic modality for hair loss, offering a potentially less invasive and biologically driven approach. Due to the high clinician and consumer interest in this type of therapy, many CE-marked preparation kits have been marketed. However, important variability characterizes these kits and the end-product which they derive, affecting the quality and efficacy of the obtained biological product.

The PRP treatment procedure commences with a venipuncture to collect a volume of whole blood, typically ranging from 10 to 60 mL or even 100 mL, which is immediately mixed with an anticoagulant to prevent premature platelet activation [23,26,29]. Subsequently, the blood undergoes a series of centrifugation steps to separate red blood cells from the plasma, followed by a final centrifugation step to isolate the platelet-rich plasma from the platelet-poor plasma. The composition of the final PRP product can be tailored by selectively collecting a portion of the PRP along with residual red blood cells, depending on the desired therapeutic outcome. Activation of the PRP is then achieved using calcium chloride or thrombin, triggering platelet activation and fibrin formation. This process yields a highly concentrated PRP preparation, with platelet levels typically 2 to 8 times greater than those found in whole blood [27]. Thus, despite the initial need for large amounts of blood, the final PRP volume to be used is often between 2 and 5 mL.

The administration of PRP involves injecting the prepared solution into the scalp using a fine hypodermic needle (30–32 G). However, this procedure can be associated with discomfort and potential bleeding, necessitating the use of surface anesthesia [23,26,28,30]. Furthermore, the requirement for fresh preparation immediately before use and physician administration raises logistical considerations regarding its clinical applicability. Specifically, the necessity of a medical professional increases the cost and reduces access to the treatment.

Despite its potential, the widespread adoption of PRP in clinical practice is hampered by several constraints. Inconsistent preparation protocols across institutions and a lack of standardized reporting in the literature contribute to variability in PRP quality and efficacy [26,28]. In recent years, several authors have attempted to address the challenges associated with interpreting and comparing data from PRP studies by proposing standardized reporting frameworks. Fadadu et al. (2019) emphasized the essential parameters that should be consistently reported in clinical PRP studies, focusing both on the characteristics of the final PRP product and on the preparation protocol [31]. Similarly, Everts et al. (2020) [27] proposed key elements for the development of a PRP classification system. When comparing these reviews, overlapping parameters emerge as minimum methodological requirements for clinical reporting. These include, for the final PRP product, platelet concentration and the presence or absence of leukocytes (i.e., allowing classification into leukocyte-rich PRP [L-PRP] versus pure PRP [P-PRP]) and, for the preparation protocol, centrifugal force and duration (i.e., *g*-force and time), number of spin cycles, and the use of activators.

With specific regard to hair restoration, a 2025 systematic review and meta-analysis encompassing 43 clinical studies evaluated the efficacy of PRP in alopecia management [32]. The analysis concluded that PRP represents a generally safe and effective therapeutic option for alopecia, demonstrating consistent increases in hair density and reductions in recurrence rates compared with placebo. Notably, activated PRP appeared to produce more favorable outcomes, whereas non-activated PRP was associated with a higher incidence of adverse effects [32]. However, substantial heterogeneity in study design, PRP preparation methods, and incomplete reporting of product composition limited the ability to detect clear differences in efficacy among alopecia subtypes [32]. These findings further underscore the importance of standardized preparation protocols and comprehensive methodological reporting to improve reproducibility and interpretability in PRP research. To illustrate this heterogeneity, Table 1 provides a comparative overview of representative clinical studies on PRP in hair loss, highlighting substantial variations not only in preparation protocols, dosing, and injection parameters, but also in the PRP preparation kits supplied by different manufacturers, which directly influence platelet concentration, activation status, and final product characteristics. Additionally, patient biological differences, such as age, health status, and lifestyle, can influence the composition and effectiveness of PRP, further complicating its clinical application.

Recent research has also focused on optimizing PRP preparation protocols, investigating the use of different commercial kits, centrifugation techniques, and activation methods to enhance growth factor release and improve clinical outcomes [28]. Furthermore, studies are exploring the synergistic effects of combining PRP with other hair loss treatments, such as microneedling, single hair transplants, or laser therapy, to maximize therapeutic benefits. Overall, the future of PRP therapy lies in the standardization of preparation protocols, the optimization of delivery methods, and the integration of evidence-based practices to ensure consistent and reliable clinical outcomes.

### 2.2. Use of Stem Cells for Hair Loss Management

The field of regenerative medicine has witnessed increasing interest in stem cell-based therapies for hair restoration, driven by their potential to modulate the hair follicle microenvironment and stimulate follicular regeneration [38,39,40,41,42,43]. These approaches aim to activate hair follicle stem cells (HFSCs) and dermal papilla cells, thereby promoting transition into the anagen phase and supporting follicular cycling. Stem cell-based strategies in hair restoration can be broadly categorized according to their cellular source into autologous and allogeneic approaches, as discussed hereafter.

#### 2.2.1. Autologous Stem Cell-Based Therapies

Autologous stem cell-based interventions in hair restoration involve the isolation and re-administration of patient-derived cellular fractions, most commonly adipose-derived stem/stromal cells (ADSCs), stromal vascular fraction (SVF), or scalp-derived micrografts enriched in hair follicle stem cells (HFSCs) [38,39,40,41,42,43,44]. These approaches eliminate risks associated with alloimmunization and donor-derived variability while potentially simplifying regulatory classification when cells are minimally manipulated.

Adipose tissue remains the predominant source for autologous regenerative applications owing to its relative abundance, minimally invasive harvesting via liposuction, and significantly higher frequency of mesenchymal progenitor cells compared with bone marrow–derived sources [45,46,47]. Two principal preparation strategies are typically employed: enzymatic digestion to isolate the SVF, and purely mechanical microfragmentation techniques. Enzymatic processing using collagenase yields a heterogeneous stromal population containing ADSCs, pericytes, endothelial progenitors, and immune cells [45,47]. However and importantly, enzymatic digestion is widely classified as more-than-minimal manipulation under U.S. FDA (21 CFR 1271) and European Medicines Agency (EMA) regulatory frameworks [48]. In contrast, mechanical processing techniques that preserve tissue architecture without enzymatic disruption may comply with minimal-manipulation criteria depending on jurisdictional interpretation [48].

Clinically, most published data consist of prospective case series, small cohort studies, and a limited number of controlled trials evaluating autologous adipose-derived cellular therapies for AGA [41,49,50]. Reported outcomes generally show increases in hair density of approximately 10–50% from baseline at 3 to 6 months, along with improvements in hair shaft thickness, although the magnitude of these effects varies considerably between studies [41,49,50]. Specifically, methodological heterogeneity remains considerable. Variability in cell isolation techniques (e.g., enzymatic SVF vs. mechanical micrografts), injected cell counts, treatment intervals, and outcome assessment tools (e.g., phototrichogram, standardized global photography, trichoscopy, or investigator global assessment) complicates cross-study comparison and limits meta-analytic interpretability [41,49,50]. Randomized controlled trials remain scarce, and few studies incorporate placebo or active comparators. Most cohorts are small (frequently <50 patients), with follow-up durations typically restricted to 6–12 months [41,49,50]. Long-term durability beyond 12–18 months has rarely been systematically evaluated, and repeat-treatment strategies are not standardized. Importantly, female-pattern hair loss (FPHL) remains underrepresented in published trials, limiting extrapolation of findings to this population and highlighting a persistent evidence gap [41,44,49,50]. Reported adverse events across autologous studies are generally mild and transient, consisting primarily of injection-site pain, erythema, edema, ecchymosis, and occasional transient scalp tenderness [41,49,50]. To date, no cases of tumorigenesis have been reported in clinical hair restoration cohorts, however, systematic long-term oncologic surveillance is lacking, and safety reporting remains inconsistent across studies. Of note, the absence of standardized adverse-event grading and extended follow-up limits drawing definitive conclusions regarding long-term risk profiles [41,49,50].

#### 2.2.2. Allogeneic Stem Cell-Based Therapies

Allogeneic stem cell-based strategies for hair restoration utilize donor-derived mesenchymal stromal/stem cells (MSCs), culture-expanded cell populations, conditioned media, or extracellular vesicle-enriched preparations (often marketed as exosome-based therapies) [45,51]. These approaches aim to capitalize on the paracrine regenerative capacity of MSCs while enabling scalable manufacturing and product standardization [52].

Unlike autologous preparations, allogeneic products require in vitro expansion, phenotypic characterization, and quality control prior to clinical use. Culture expansion and ex vivo manipulation generally constitute substantial manipulation under FDA and EMA regulatory definitions, placing these products within advanced therapy medicinal product (ATMP) or biologics frameworks and requiring stringent manufacturing oversight [48,53].

Mechanistically, allogeneic MSCs and MSC-derived products act primarily through paracrine signaling rather than durable engraftment. Secreted growth factors (e.g., VEGF, HGF, IGF-1), cytokines, and EV-associated microRNAs modulate Wnt/β-catenin signaling, dermal papilla cell activity, angiogenesis, and local immune regulation within the follicular niche [39,54]. To the best of our knowledge, there is currently only one randomized, double-blind, placebo-controlled clinical trial evaluating allogeneic mesenchymal stem cells (i.e., bone marrow MSCs) in AGA, and it was conducted exclusively in male patients [54]. While this study reported statistically significant short-term improvements in hair count and diameter compared with placebo, the evidence remains limited to a single-center experience with restricted follow-up duration [54]. No randomized controlled data are presently available in female-pattern hair loss, and long-term durability and safety beyond the short reported observation period remain insufficiently characterized. Generally, allogeneic stem cell-based therapies for hair loss should currently be considered investigational. Robust randomized controlled trials with extended follow-up, standardized product characterization, and inclusion of female-pattern hair loss cohorts are required to define their true clinical efficacy, durability, and safety profile.

### 2.3. Use of Exosomes for Hair Loss Management

Exosomes, nano-sized extracellular vesicles (EVs) ranging from 40 to 200 nm in diameter, have emerged as pivotal mediators of intercellular communication, orchestrating the transport of diverse cellular materials between cells [55,56,57,58,59,60,61,62,63,64]. These vesicles serve as conduits for bioactive molecules, including cytosolic proteins, enzymes, transcription factors, extracellular matrix proteins, receptors, and nucleic acids such as mRNA, miRNA, and DNA, facilitating the transfer of biological information and functional modulation of recipient cells [56,57,61,62,63,64]. Initially perceived as cellular waste products, exosomes were first identified in the 1980s. However, subsequent research has unveiled their critical roles in a myriad of biological processes, including immune responses, cell growth, and tissue regeneration [56,62]. Moreover, their involvement in the pathogenesis of various diseases, such as cancer, neurodegenerative disorders, and cardiovascular diseases, has opened new avenues for understanding disease mechanisms and exploring innovative therapeutic strategies [56,63]. For clarity, it is important to distinguish between terms frequently used in the field of hair loss management and aesthetics, which do not always adhere to the Minimal Information for Studies of Extracellular Vesicles (MISEV2023) guidelines [65]. “Extracellular vesicles” (EVs) is the preferred generic term for lipid-bilayer particles released by cells that cannot replicate independently, encompassing subtypes such as exosomes and microvesicles defined by their biogenesis and size range [55,56,57,58,59,60,61,62,63,64,65]. “Secretome” or “conditioned media” refers more broadly to the full mixture of soluble factors and vesicular elements secreted by cultured cells, without selective purification of EV subpopulations. The term “exosome-mimetic” or “EV-based preparation” is often applied to engineered or proprietary products that do not necessarily reflect endogenous EV biogenesis or meet minimal characterization criteria [55,64,65,66,67]. In many commercially available hair restoration products, detailed characterization according to MISEV2023 recommendations (e.g., particle size/concentration, canonical EV markers such as CD9/CD63/CD81, morphology, and explicit isolation methods) is not publicly disclosed, justifying use of the more neutral designation “EV-based preparations” when reporting clinical outcomes [65].

In the context of hair loss, exosomes derived from various cell types (i.e., from human, animal, or plant) have emerged as a promising cell-free regenerative approach. A systematic review by Al Ameer et al. in 2025 analyzed clinical evidence across 11 studies involving exosomes sourced from adipose tissue, placenta, foreskin, hair follicles, bone marrow, and umbilical cord [68]. Across alopecia types, including mainly AGA but also chemotherapy-induced alopecia, exosome-based interventions consistently demonstrated improvements in hair density, hair shaft thickness, and overall scalp coverage [68]. These exosomes exert their effects through complex mechanisms involving microRNAs (miRNAs), which modulate key signaling pathways, including the Wnt/β-catenin and BMP pathways, essential for regulating dermal papilla cell (DPC) activity and promoting hair follicle stem cell proliferation [56,58,64]. Research suggests that exosome-mediated delivery of miRNAs can effectively shift hair follicles from the resting (telogen) to the active (anagen) phase, thereby promoting hair regrowth and addressing hair loss caused by immune-related factors [61].

Both topical and injectable exosome therapies have shown promising results in preclinical and early clinical studies [68]. However, a consensus on the optimal administration method of exosomes remains elusive. The choice between these approaches may vary depending on the clinical context, the exosome source, and the desired therapeutic outcome. Nonetheless, transdermal drug delivery is gaining preference due to its ability to bypass the stratum corneum, enhance the skin’s absorption of active compounds, and minimize the risk of systemic side effects [56,63]. The use of microneedling in conjunction with topical exosome application is also showing promise [68].

However, despite encouraging preliminary outcomes, the clinical application of exosome-based therapies for hair loss is still in its nascent stages. Comprehensive clinical trials are imperative to validate their effectiveness, safety, and long-term advantages [62,64]. Moreover, several challenges need to be addressed to facilitate the translation of exosome-based therapies from bench to bedside. Preparations differ in biological source (human, animal, or plant), donor screening procedures, culture conditions, isolation techniques (e.g., ultracentrifugation, precipitation, size-exclusion chromatography), purification stringency, formulation (lyophilized versus liquid), and administered dose. Importantly, critical quality attributes, including particle concentration, size distribution, purity indices, sterility, and potency, are rarely standardized or uniformly reported. To enhance translational rigor, a minimal characterization framework for clinical-grade exosome preparations should include: GMP-compliant manufacturing and batch traceability, explicit declaration of biological origin and donor screening (i.e., for human-derived products), transparent isolation methodology, physicochemical characterization (e.g., particle size and concentration, canonical markers such as CD9, CD63, CD81, and purity ratios), sterility and endotoxin testing, and at least one validated potency surrogate relevant to hair biology (e.g., dermal papilla proliferation or Wnt/β-catenin activation assays). Stability data and defined storage conditions should also be documented. Such variability in commercially-sourced products compromises batch-to-batch consistency, a fundamental requirement for clinical translation and inter-study comparability [56,65,68]. The absence of standardized protocols for characterizing and quantifying exosomes further exacerbates the challenges related to reproducibility and reliability.

Furthermore, concerns regarding off-target effects and unintended biological responses remain. While exosomes are generally considered less immunogenic than whole cells, they can still elicit immune reactions, particularly when derived from allogeneic cells. The diverse array of bioactive molecules contained within exosomes may also have unpredictable effects on recipient cells, raising safety concerns related to proinflammatory or oncogenic risks [64]. Stringent safety assessments and long-term monitoring are essential to mitigate these risks. The scalability of exosome production is another critical consideration for clinical translation. Current production methods often rely on small-scale cell culture techniques, which may not be feasible for large-scale manufacturing. The development of scalable and cost-effective production platforms is essential to ensure the widespread availability of exosome-based therapies [69]. In addition, these preparations will be strictly controlled from a regulatory standpoint, with increasing pressure to standardize manufacturing processes and categorize products as drugs or biologicals. Of note, there are currently no injectable FDA-approved exosome products [55,70].

In the context of hair regrowth, several topical exosome-based products are currently available, differing in biological source and pharmaceutical form. ASCEplus^®^ HRLV (ExoCoBio, Seoul, Korea) is among the most frequently cited products and is based on plant-derived exosomes, supplied in a freeze-dried formulation suitable for scalp application [71]. Other Korean products, such as Plenaris HGF (Nexus Pharma, Seoul, Korea), are likewise provided in lyophilized form and rely on umbilical cord-derived MSC exosomes [72]. From Switzerland, Exovyal^®^ (Louna Regenerative/Louna Aesthetics, Geneva, Switzerland) is formulated as a freeze-dried exosome-based product, relying on plant-derived/or human-derived exosome sources, and is also positioned for hair stimulation [73]. In contrast, European products, particularly from Spain, including SKINDERMA Medical Cosmetics (Huesca, Spain) and MCCM Medical Cosmetics (Barcelona, Spain), predominantly offer liquid exosome or EV formulations intended for hair and scalp applications [74,75]. Other products using terms such as “synthetic exosomes” or “biosomes” do exist, however, to date, there is a lack of publicly available information and supporting scientific publications. 

At present, no universally accepted clinical-grade standards exist for exosome therapies in aesthetic or hair restoration applications. Although early clinical studies and systematic reviews report improvements in hair density and thickness [68], the evidence is largely derived from small, non-randomized cohorts with short follow-up durations and inconsistent safety reporting. Well-designed randomized controlled trials with standardized product characterization and structured pharmacovigilance are therefore required before widespread clinical adoption can be recommended.

Future research should focus on optimizing exosome production and isolation methods, developing standardized characterization and quantification protocols, and conducting rigorous preclinical and clinical studies to evaluate the safety and efficacy of exosome-based therapies for hair loss. By addressing these challenges, exosomes hold immense potential to revolutionize hair restoration and provide effective and safe therapeutic options for individuals affected by hair loss.

### 2.4. Use of Intradermal Botulinum Toxin for Hair Loss Management

Botulinum toxin type A (BTA), a potent neurotoxin produced by *Clostridium botulinum*, exerts its effects by modulating the release of acetylcholine at neuromuscular junctions and other cholinergic synapses [76,77]. In dermatology, BTA is widely utilized for a spectrum of applications, including the reduction in rhytides, modulation of facial muscle activity, treatment of hyperhidrosis, correction of masseter hypertrophy, and management of gastrocnemius hypertrophy [76,77,78,79,80,81,82,83,84]. While BTA has demonstrated promising potential in the treatment of AGA, the precise mechanisms underlying its effects on hair follicles remain to be fully elucidated [78,85]. The proposed mechanisms of action for BTA in AGA involve its ability to inhibit acetylcholine release, a neurotransmitter crucial for nerve-muscle communication. By inducing relaxation of the scalp musculature, BTA may enhance microcirculation and oxygen delivery to the affected areas, potentially mitigating hair thinning [78,80,81,83,84,85,86,87]. Experimental and clinical observations suggest that reduction in perifollicular vascular compression may enhance tissue perfusion and reduce local hypoxia, which has been implicated in DHT-mediated miniaturization [88]. Furthermore, BTA may exert inhibitory effects on DHT, a key mediator of AGA, thereby further reducing hair loss [78,81,85,86,87]. The increased oxygen concentration in the scalp may also stimulate hair follicles, promoting the transition from the telogen to the anagen phase and fostering hair regeneration [84].

Of note, TGF-β1 has been implicated in the pathogenesis of AGA, as it inhibits the proliferation of follicular keratinocytes and disrupts the hair growth cycle. BTA has been shown to reduce TGF-β1 secretion from DPCs in vitro, potentially preventing the progression of AGA [78,80,82,83,87,89]. Additionally, BTA may modulate the production of proinflammatory cytokines and influence the interplay between hair follicle cells and immune cells, potentially creating a more favorable environment for hair growth [78,85].

Clinical evidence remains limited and methodologically heterogeneous. A systematic review identified five clinical studies including 165 predominantly male participants, reporting increases in hair count of approximately 18–21% from baseline, with response rates around 75–79% [88]. A recent a meta-analysis (i.e., 2025) reported statistically significant improvements in hair density compared with controls, but substantial heterogeneity, small sample sizes, and variable dosing and injection protocols were noted [90]. Importantly, a 2025 randomized controlled trial in 15 men with AGA comparing subcutaneous injection versus combined subcutaneous and intramuscular administration (100 units of toxin in total) found no statistically significant improvement in hair density, vellus-to-terminal hair ratio, or global photographic assessment at 6 months in either group. No superiority of combined intramuscular delivery was demonstrated, and a reduction in hair thickness was observed in the frontal region in both arms [91]. These findings underscore the variability of outcomes and the need for cautious interpretation of earlier uncontrolled or non-blinded studies. Several methodological limitations characterize the current literature. Most studies are small, frequently enrolling fewer than 50 patients, and include predominantly male cohorts, limiting extrapolation to female-pattern hair loss [88,90]. Follow-up duration is typically short (i.e., ≤6 months), which is particularly problematic given seasonal variations in hair cycling and the known placebo effect in alopecia trials [88]. Furthermore, primary endpoints are inconsistently defined, while some studies rely on global photography or subjective improvement scales, objective measures such as phototrichogram-based hair density, quantitative hair shaft diameter assessment, and validated visual grading scales are not uniformly prespecified [88,91]. Comparative trials against established therapies such as minoxidil or finasteride are lacking, and durability beyond 6–12 months has rarely been systematically evaluated [90].

Injection protocols also vary considerably, targeting the frontalis, temporalis, occipital, and periauricular musculature, with total doses ranging from approximately 50 to 150 toxin units per session [88]. Subcutaneous scalp injections have likewise been explored. However, given the absence of consistent superiority between injection depths and the heterogeneity of dosing regimens demonstrated in recent comparative data, these injection maps should be regarded as provisional and hypothesis-generating rather than standardized therapeutic protocols [91]. Despite promising preliminary findings, several critical limitations thus exist in the current body of research on botulinum toxin injections for AGA. One major concern is the paucity of robust clinical data to draw definitive conclusions regarding its efficacy. The absence of control groups in the reviewed studies is particularly problematic, as clinical trials for hair loss often exhibit a significant placebo effect, particularly in subjective assessments and hair counts. Seasonal fluctuations in scalp hair counts can further confound the interpretation of results, especially in short-term studies lacking control groups. Age may also play a significant role in the response to botulinum toxin, as younger individuals tend to have stronger muscles, potentially requiring higher doses for optimal results. Studies that fail to account for age-related differences may introduce bias into the outcomes. Furthermore, the limited representation of female participants in most studies restricts the generalizability of the findings to women with pattern hair loss.

Another critical gap in the current research is the lack of comparative studies evaluating the efficacy of botulinum toxin injections against FDA-approved AGA treatments, such as finasteride or minoxidil. This omission leaves it unclear whether botulinum toxin offers comparable or superior efficacy to standard therapies. Finally, the absence of assessments on changes in hair diameter represents a significant limitation, as hair follicle miniaturization is a hallmark feature of AGA [88]. Understanding the impact of botulinum toxin on hair diameter is crucial for evaluating its potential to reverse or prevent follicular miniaturization. Future research should prioritize well-designed, randomized controlled trials with adequate sample sizes, including both male and female participants, and follow-up periods extending beyond 12 months to clarify efficacy, durability, and optimal dosing strategies to address these limitations and provide a more comprehensive understanding of the therapeutic potential of botulinum toxin in AGA.

### 2.5. Use of Hair Boosters for Hair Loss Management

**Figure 2:**
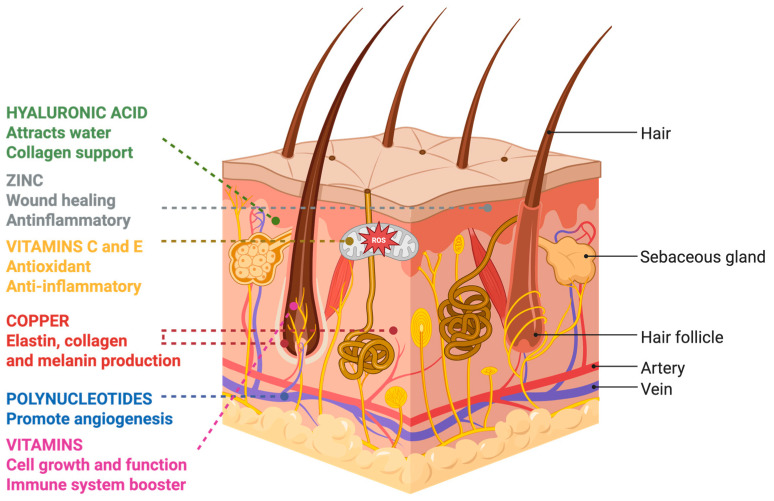
Activity of the key ingredients in commercially available hair boosters. Hyaluronic acid promotes tissue hydration, collagen production and reinforces the intercellular structures [95]; zinc promotes wound healing and reduces inflammation [96]; vitamins C and E have antioxidant and anti-inflammatory effects [97]; copper upregulates collagen, elastin fiber components, and melanin biosynthesis [98,99]; polynucleotides stimulate the secretion of VEGF, which stimulates the formation of new blood vessels [100]; vitamins support cell growth and function and strengthen the immune system [101].

Hair boosters represent a diverse category of topical or injectable formulations designed to stimulate hair regrowth and improve hair health. Although often grouped under a single term, these products differ substantially in composition, biological mechanism, regulatory classification, and level of clinical evidence. A more appropriate framework is to categorize them according to their principal active component, most commonly hyaluronic acid (HA)-based formulations, polynucleotides (PN)- or polydeoxyribonucleotide (PDRN)-based formulations, or multicomponent formulations (e.g., peptide-, vitamin-, and micronutrient-based) (Table 2, Figure 2) [92,93,94].

The rationale behind hair boosters lies in the synergistic effects of these ingredients, which collectively target various aspects of hair follicle biology and scalp health. When administered via injection, mesotherapy, or in conjunction with microneedling, the active constituents of hair boosters can penetrate deeper into the scalp, bypassing the stratum corneum and maximizing their therapeutic effects [102]. This enhanced delivery method allows for a more direct interaction between the bioactive molecules and hair follicle stem cells, dermal papilla cells, and other relevant cellular components, thereby promoting hair growth and regeneration.

The regulatory landscape surrounding hair boosters varies depending on their classification and intended use. Class III medical devices containing HA or PN for hair regrowth face stringent regulatory hurdles, necessitating rigorous clinical trials and safety assessments before market approval [103]. Conversely, hair boosters classified as non-invasive cosmetic products, subject to less stringent approval processes, are more readily accessible to consumers. This regulatory disparity reflects the differing levels of risk associated with these product categories.

#### 2.5.1. Hyaluronic Acid-Based Hair Boosters

Hyaluronic acid (HA), a naturally occurring glycosaminoglycan found in the connective tissues of various organisms, plays a crucial role in maintaining tissue hydration, elasticity, and structural integrity [104,105,106,107]. Its presence in the vitreous humor, synovial fluid, and extracellular matrix underscores its diverse biological functions. Cross-linked HA has gained widespread acceptance in various facial aesthetic procedures, including eyebrow and labial fold shaping and volumization, owing to its ability to provide structural support and enhance tissue volume [104,108,109,110]. In scalp applications, however, linear (non-cross-linked) HA is more commonly employed. Unlike cross-linked fillers designed for volumization, non-cross-linked HA is primarily used as a bio-revitalizing matrix or carrier vehicle, allowing incorporation of amino acids, vitamins, peptides, and active principles. Its main physicochemical contribution is the increase in formulation viscosity and dermal residence time, potentially improving local diffusion kinetics and sustained exposure of follicular structures to co-administered actives. Preclinical data support a biologically plausible role of non-cross-linked HA in modulating follicular biology. In vitro experiments using human dermal papilla cells (HDPCs) demonstrated that a non-cross-linked HA formulation enriched with amino acids increased cell viability under oxidative stress conditions and significantly enhanced VEGF secretion, suggesting a pro-angiogenic and cytoprotective effect [111]. In animal models of AGA, HA liposomes have been investigated as drug delivery vehicles for minoxidil, demonstrating their ability to enhance drug penetration and prolong drug release [112,113,114,115]. A recent clinical study evaluating a stabilized, mechanically processed non-cross-linked HA formulation supplemented with vitamins, amino acids, and ions (i.e., CELLBOOSTER^®^ Hair) in 26 adults with moderate AGA reported significant increases in hair thickness, density, and shine, with excellent tolerability and a majority of subjects reporting improved appearance over a 90-day follow-up period [113]. While intriguing, this evidence originates from a single cohort study without placebo control, and objective measures such as phototrichogram-based density or quantitative hair diameter were not uniformly prespecified. However, paradoxical cases of alopecia have also been reported following HA injections, highlighting the need for careful patient selection and administration techniques [116,117,118,119,120,121,122].

The conflicting reports regarding the effects of HA on hair growth underscore the complexity of its interactions with hair follicle biology. While some studies have documented adverse effects, others have demonstrated the beneficial effects of HA-based compounds in promoting hair regrowth and improving hair quality [111,123,124]. These beneficial effects may be attributed to HA’s ability to enhance tissue hydration, promote angiogenesis, and stimulate the proliferation of dermal papilla cells. Furthermore, HA-based hair boosters often incorporate other bioactive ingredients, such as peptides and growth factors, which can synergistically enhance the effects of HA (Table 2). Taken together, HA-based scalp therapies are biologically plausible and may contribute to microenvironmental modulation through hydration support, extracellular matrix effects, and carrier-mediated delivery of adjunctive compounds. However, robust, well-controlled randomized trials with standardized formulations and objective endpoints are needed to determine whether HA itself, independent of co-administered ingredients, confers clinically meaningful benefit in hair loss management.

#### 2.5.2. Polynucleotide-Based Hair Boosters

Polynucleotides (PNs) are highly purified DNA-derived macromolecules, most commonly extracted from salmonid sources, that have been introduced in aesthetic dermatology as bio-stimulatory injectable agents with regenerative potential [100]. Their proposed mechanisms include modulation of adenosine A2A receptor signaling, upregulation of angiogenic mediators such as VEGF, improvement of microcirculation, and anti-inflammatory effects through regulation of cytokine pathways. Adenosine promotes hair growth and inhibits the catagen transition in hair follicles by stimulating the Wnt/β-catenin signaling pathway and enhancing the expression of growth factors. Collectively, these mechanisms may create a follicular microenvironment supportive of anagen induction and hair shaft strengthening [125,126,127].

Clinical data in AGA remain limited but are gradually emerging. A prospective clinical investigation evaluating intradermal PN injections over four treatment sessions reported statistically significant increases in hair shaft diameter and total hair density, with high patient satisfaction and no serious adverse events [125]. Similarly, a 24-week study assessing a PN-based gel formulation administered in repeated intradermal sessions demonstrated improvements in trichoscopic parameters, including hair thickness and terminal-to-vellus hair ratio, with favorable tolerability [128]. A small comparative study evaluating a PN gel against PRP suggested broadly comparable short-term improvements [129]. Similarly, a study comparing PDRN and PRP injection in treating female pattern hair loss has showed that combined therapy with PRP and PDRN induces greater improvement in hair thickness than treatment with PDRN therapy alone (*p* = 0.031), but not in hair counts (*p* > 0.05) [130]. However, the absence of robust randomization, small sample size, and incomplete long-term follow-up limit interpretation.

Despite these encouraging findings, the current body of evidence remains characterized by modest cohort sizes, short observation periods (i.e., typically ≤6 months), heterogeneity in injection protocols, and a predominance of non-randomized designs. Objective endpoints such as standardized phototrichogram-based hair density and quantitative shaft diameter measurements are not uniformly prespecified across studies. Furthermore, long-term durability beyond the initial treatment cycle has not been systematically evaluated. Therefore, while PN-based therapies appear biologically plausible and clinically promising, the available data are insufficient to establish definitive efficacy, and adequately powered randomized, placebo-controlled trials with standardized outcome measures and extended follow-up are required to clarify their therapeutic role in AGA.

#### 2.5.3. Multicomponent Formulations Used as Hair Boosters

Multicomponent formulations (e.g., peptide-, vitamin-, and micronutrient-based) represent a heterogeneous category of topical or mesotherapy scalp preparations marketed for hair revitalization. These products typically combine biomimetic peptides, B-complex vitamins (e.g., biotin, pyridoxine), antioxidants (e.g., vitamins C and E), amino acids, and zinc or copper. From a biological standpoint, peptides are proposed to act as signaling molecules that may influence dermal papilla cell activity, while vitamins and micronutrients primarily serve as metabolic cofactors involved in keratin synthesis and cellular oxidative balance [97,131,132,133]. Clinical evidence for multicomponent mesotherapy in telogen effluvium remains limited and is primarily derived from small randomized comparative studies. In a trial including 24 female patients, participants received either intramuscular botulinum toxin A or multivitamin mesotherapy. The mesotherapy group was treated with a multivitamin cocktail containing water, minerals, vitamins B, A, E, D, and C, dexpanthenol, caffeine, saw palmetto, *Ginkgo biloba*, cysteine, methionine, taurine, biotin, and zinc. At each session, 2 mL was injected intradermally across the scalp using a nappage technique, with injection points spaced 1 cm apart and 0.05 mL delivered per site using an insulin syringe. Injection depth ranged from 2 to 4 mm at an angle of 30 to 60 degrees. Treatments were administered every two weeks for two months, followed by monthly maintenance sessions for four additional months. Both groups demonstrated improvements in trichoscopic parameters at 3 and 6 months, with no statistically significant difference found between groups in most objective outcomes. Reported adverse events were mild and mainly limited to injection-related pain. An advantage of botulinum toxin in this study was the single-session administration, compared with the multiple treatment sessions required for mesotherapy [133]. However, most supporting data are derived from in vitro studies or theoretical mechanistic rationale rather than robust clinical trials.

These multicomponent formulations are highly variable in composition, concentration, and administration protocols (e.g., mesotherapy, microneedling-assisted delivery), which significantly limits comparability across studies. In many regulatory settings, such preparations are positioned and regulated as cosmetic or aesthetic adjunct products rather than therapies. Objective endpoints were variably defined, and placebo-controlled data as well as long-term durability remain lacking. Consequently, although these formulations are currently widely used in aesthetic practice, their independent efficacy has not been definitively established.

Notably, the variability in hair booster formulations and administration methods underscores the need for standardized protocols and rigorous clinical trials to evaluate their safety and efficacy. Future research should focus on elucidating the precise mechanisms by which hair boosters promote hair growth, optimizing their formulations and delivery methods, and conducting comparative studies to evaluate their efficacy against established hair loss treatments, even if the margin of improvement is relatively narrow. Additionally, specific studies are needed to evaluate the long-term safety and efficacy of these boosters.

### 2.6. Levels of Evidence for the Described Interventions

According to the Oxford Centre for Evidence-Based Medicine (OCEBM) 2011 Levels of Evidence for treatment benefits, PRP has the most mature clinical evidence base among the injectable modalities discussed in this review [134]. Multiple randomized controlled trials and several systematic reviews/meta-analyses support the use of PRP, which corresponds overall to Level 1 evidence, although the certainty may be graded down because PRP preparation protocols, dosing, and endpoints are highly heterogeneous across studies and often incompletely reported (e.g., differences in kits, activation, platelet/leukocyte content, and injection parameters). For botulinum toxin, the evidence base includes systematic reviews/meta-analyses and a small number of randomized trials, which places the modality broadly in the Level 1–2 range by design, however, confidence remains limited because studies are few, small, and heterogeneous, and at least one recent randomized clinical trial reported no significant benefit with outcomes varying across endpoints and protocols [91]. For stem cell-based approaches, the overall body of evidence remains earlier-stage than PRP. Autologous preparations (e.g., SVF/ADSC-derived cellular fractions and micrografts) are mainly supported by small prospective cohorts, case series, and a limited number of controlled studies summarized in systematic reviews, which corresponds most consistently to Level 3 evidence in practice (i.e., despite the existence of systematic reviews, the underlying trials are sparse and underpowered). Allogeneic stem cell–derived products and conditioned media are supported mainly by early-phase and uncontrolled clinical investigations, placing them at Level 3–4 evidence overall; to date, only one randomized double-blind placebo-controlled trial has been reported and it was conducted exclusively in men with AGA. Exosome and EV-based therapies are supported by systematic reviews summarizing small prospective cohorts and early-phase studies. However, randomized controlled trials remain scarce, product characterization is inconsistent, and adherence to MISEV2023 guideline criteria is variable. Accordingly, exosome/EV-based interventions currently correspond to Level 3–4 evidence, with confidence limited by small sample sizes, short follow-up, and substantial variability in biological source, manufacturing, and reporting standards. Finally, “hair boosters” (i.e., HA-based formulations, polynucleotides/PDRN, and multicomponent mesotherapy cocktails) are supported mostly by small prospective studies, case series, and limited comparative trials, placing them predominantly at Level 3–4 evidence. Taken together, while several interventions show biological plausibility and early clinical signals, PRP is the only modality in our reference set that consistently approaches higher-tier evidence under the OCEBM 2011 framework, whereas the remaining modalities should be interpreted with moderate-to-low confidence pending larger, methodologically standardized randomized trials with longer follow-up and stronger safety reporting.

## 3. Scientific Relevance of Recent Clinical Studies and Methodological Assessments

The landscape of AGA management is characterized by a paradox: while numerous treatments are widely employed in clinical practice, many lack rigorous scientific validation regarding their safety and efficacy specifically for AGA and rely on tenuous marketing promises. This disparity underscores a critical need for robust clinical trials to generate reliable evidence-based data, thereby informing clinical decision-making and enhancing patient outcomes. Although many treatments are considered to be generally safe in other medical contexts, they can still elicit significant adverse effects when applied to AGA, further emphasizing the importance of dedicated research [50,135,136,137]. The complexities of AGA management are compounded by several factors, including the limited number of FDA-approved treatments, the proliferation of off-label options, the paucity of clinical trials for many of these off-label therapies, and the heterogeneity of patient preferences and expectations [50,68,111,124,135,136,137]. These challenges highlight the need for a comprehensive and individualized approach to AGA treatment.

The diagnosis of AGA is primarily clinical, relying on the characteristic pattern of nonscarring hair loss. A thorough physical examination, including a pull test and assessment of facial and body hair, is essential to differentiate AGA from other conditions, such as diffuse TE or AA. Trichoscopy, a non-invasive technique that visualizes hair and scalp structures, can be invaluable in ambiguous cases, revealing features such as hair shaft diameter variability, increased vellus hairs, yellow dots, and perifollicular discoloration. In situations where clinical findings are inconclusive, laboratory tests or histological examination of scalp biopsies may be warranted to confirm the diagnosis [138,139].

In clinical practice, AGA severity is typically documented using standardized pattern classifications, such as the Hamilton-Norwood scale for men and the Ludwig scale for women. However, the absence of a universally accepted definition for AGA severity and progression poses challenges in accurately assessing treatment outcomes. These classification systems often fail to capture the dynamic nature of AGA progression, which is characterized by gradual hair thinning and follicular miniaturization. Given the progressive nature of AGA, treatment goals typically focus on halting hair loss progression and promoting hair regrowth. However, the evaluation and monitoring of hair growth are often subjective, relying on patient and physician assessments. To enhance objectivity in clinical studies, standardized objective methods are employed, including hair count/density measurements and global photographic assessments. Hair counts and density measurements provide quantitative data on hair follicle density and distribution, while global photographic assessments, evaluated by experts blinded to treatment and time, offer a semi-objective measure of overall hair growth [140,141].

Advanced digital imaging systems have emerged as valuable tools for quantifying hair density, thickness, and the ratio of different hair types within a defined scalp area, often marked with a tattoo to ensure consistent measurement locations. These systems provide precise and reproducible data, enhancing the reliability of clinical trial outcomes. For effective evaluation in clinical trials, comparisons to baseline, placebo, or other active treatments are essential to establish the efficacy of the investigational therapy. Global photographic assessment has been recognized as a particularly effective method for evaluating hair growth, as it provides a comprehensive and standardized approach to assessing the entire scalp, minimizing the influence of personal biases. This method allows for the visualization of overall hair coverage and the identification of subtle changes in hair density and distribution. Artificial intelligence tools are increasingly used by both patients and clinicians, offering future opportunities for more accurate diagnosis and personalized treatment in dermatology. Gupta et al. (2022) show that automated hair detection systems, density/diameter measurements, and deep learning scalp analysis models can standardize alopecia evaluation and improve reproducibility [142]. Image based large language models have also demonstrated strong diagnostic capacity in acne, rosacea, and hidradenitis suppurativa [143,144]. Similar protocols could easily be adapted to alopecia by using standardized scalp images to generate objective metrics and guide individualized care.

In addition to evaluating clinical efficacy, assessing the cost-effectiveness of AGA treatments is crucial for informing patient discussions and influencing treatment decisions. Given that patients often bear the full cost of treatment, it is essential to consider the benefits that are most relevant to their individual needs and preferences. Cost-effectiveness analyses can provide valuable insights into the economic impact of different treatment strategies, helping patients make informed decisions about their care [50,52,69,136]. Future clinical trials should integrate cost-effectiveness analyses to provide a more comprehensive assessment of AGA treatments. Still beyond clinical efficacy, the duration and persistence of treatment effects represent critical yet insufficiently documented outcomes in AGA research [145]. For many therapeutic modalities, including injectable treatments, the available data primarily describe short-term responses, while the long-term durability of clinical benefits remains poorly characterized. Consequently, it is unclear whether sustained clinical improvement requires continuous or repeated treatment over time, or whether certain interventions are capable of inducing a true regenerative response that maintains hair density after treatment discontinuation. Addressing this knowledge gap is essential to define optimal long-term management strategies for AGA and to distinguish maintenance therapies from approaches with the potential for durable regeneration.

Table 3 lists some of the currently ongoing clinical studies of injectable hair regrowth-promoting products.

## 4. Definition of Protocols and Techniques Used for Scalp Injections

**Figure 3:**
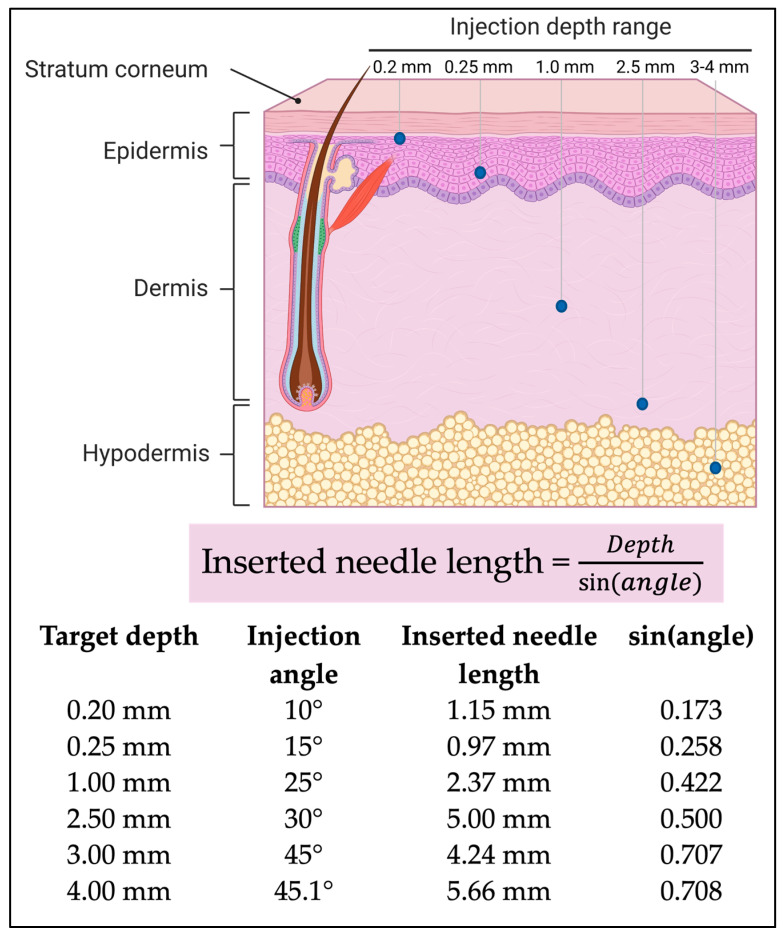
Schematic representation of skin layers (stratum corneum, epidermis, dermis, and hypodermis) illustrating the geometric relationship between injection angle and effective penetration depth. The depth of scalp injections typically ranges from approximately 0.2 mm for superficial intradermal delivery to 3–4 mm for subcutaneous injections [137]. For a given vertical depth within the tissue, the required inserted needle length increases as the injection angle decreases, following the trigonometric relationship L = Depth/sin(α), where L is the inserted needle length and α is the injection angle relative to the skin surface. This model highlights the strong influence of injection angle on depth control and the increased variability associated with shallow-angle intradermal delivery [146].

The administration of therapeutic agents via scalp injections represents a sophisticated and multifaceted approach to addressing the diverse pathologies and clinical presentations of alopecia. This method allows for targeted delivery of bioactive molecules, circumventing the limitations associated with systemic or topical treatments. The selection of an appropriate injection technique is paramount, as it directly influences the efficacy and safety of the procedure. This selection is contingent upon a thorough assessment of the specific etiology of hair loss, the severity of follicular involvement, the patient’s individual preferences and tolerance, and the characteristics of the therapeutic agent being administered (Figure 3).

For Class III medical devices containing hyaluronic acid (HA), as delineated in Table 4, which mandate medical expertise due to their inherent risk profile, intradermal injections are the predominant mode of delivery. Fine-gauge needles (30–33 G) are employed to minimize patient discomfort and tissue trauma. These injections can be administered through several distinct techniques, including micro-papule, needle microdroplet, and microneedling, each offering unique advantages in terms of drug distribution, penetration depth, and tissue interaction. Overall, the main benefits of the techniques mentioned earlier is that the therapeutic substances are delivered directly into the skin, avoiding the obstacles encountered by topical treatments, which allows for a more focused treatment approach. Consequently, it is generally believed among practitioners that the bioavailability of the substance is enhanced because it remains at the injection site for a longer duration, ensuring its direct effect on the targeted region (Table 4).

Mesotherapy and microneedling have emerged as cornerstones in the armamentarium of scalp injection techniques. Mesotherapy, a minimally invasive procedure, involves the precise intradermal injection of a customized cocktail of medicinal compounds and bioactive materials at a controlled depth, typically ranging from 2 to 4 mm [152,153]. This technique facilitates the localized delivery of therapeutic agents, minimizing systemic exposure and maximizing their concentration at the target site. The selection of injected compounds is tailored to the specific condition being treated, encompassing a broad spectrum of substances, including vitamins, minerals, amino acids, growth factors, pharmaceuticals, and polynucleotides. Recent advancements in mesotherapy have focused on optimizing the composition of injected solutions to enhance their efficacy and minimize adverse effects [154].

Microneedling, another minimally invasive technique, involves the creation of controlled micro-injuries in the skin using fine needles. This process triggers a cascade of biological responses, including the release of PDGF and VEGF, which play pivotal roles in angiogenesis, tissue repair, and scar remodeling [33,155]. In the context of hair restoration, microneedling stimulates the release of these growth factors, promoting hair follicle regeneration and potentially reversing follicular miniaturization. Furthermore, microneedling enhances the transdermal delivery of topical therapeutic agents, augmenting their efficacy by creating microchannels that bypass the stratum corneum. Recent studies have explored the synergistic effects of combining microneedling with topical growth factors, such as PRP or exosome solutions, demonstrating enhanced hair regrowth compared to monotherapy [156].

The primary advantage of these injection techniques lies in the direct delivery of therapeutic substances into the dermis, bypassing the formidable barrier posed by the stratum corneum, which significantly limits the penetration of topical treatments. This targeted delivery approach allows for a more focused and efficient treatment, maximizing the bioavailability of the therapeutic agents at the site of action. The prolonged residence time of these substances at the injection site ensures sustained interaction with target cells, enhancing their therapeutic effects and minimizing systemic side effects. This localized delivery is especially important when using potent substances that could have adverse systemic effects.

The micro-papule technique involves the creation of small, superficial wheals in the dermis, allowing for the controlled release of therapeutic agents. This technique is particularly useful for delivering small volumes of product to localized areas, such as focal AA or small areas of AGA. The needle microdroplet technique, on the other hand, involves the injection of minute droplets of product into the dermis, providing a more diffuse distribution. This technique is well-suited for treating larger areas of the scalp, such as diffuse TE or extensive areas of AGA.

Microneedling can be performed using various devices, including dermarollers and automated microneedling devices. Dermarollers, handheld devices equipped with rows of fine needles, are rolled across the scalp, creating micro-injuries. Automated microneedling devices, also known as dermapens, use reciprocating needles to create controlled punctures in the skin. The depth and frequency of needle penetration can be adjusted to suit the individual patient’s needs and the specific condition being treated, providing greater precision and control compared to dermarollers. Recent advancements in microneedling technology have focused on developing devices with enhanced precision, safety features, and patient comfort [157].

The combination of microneedling with the application of topical growth factors, such as PRP or exosome solutions, has shown promising results in hair restoration. Microneedling enhances the penetration of these growth factors into the dermis, maximizing their interaction with hair follicle stem cells and dermal papilla cells. This synergistic approach can stimulate hair follicle regeneration and promote hair regrowth. Recent studies have explored the optimal combination of microneedling parameters and growth factor concentrations to maximize therapeutic outcomes [158]. Furthermore, the integration of HA-based products with these injection techniques offers additional benefits [159].

The safety and efficacy of scalp injections are influenced by several factors, including the choice of injection technique, the selection of therapeutic agents, the expertise of the practitioner, and the patient’s individual characteristics. Proper aseptic techniques are essential to minimize the risk of infection and other complications. Patient selection is also crucial, as certain medical conditions, such as bleeding disorders or active skin infections, may contraindicate scalp injections. Pre-procedural assessments, including a thorough medical history and physical examination, are essential to identify potential contraindications and minimize the risk of adverse events. Clinicians should ensure that patients are fully informed about the nature and limits of the evidence supporting these approaches. Informed consent should explicitly disclose the off-label status of the procedure, expected benefits and uncertainties, potential risks and adverse effects, available alternative therapies, and anticipated financial costs. Standardized documentation practices, including baseline and follow-up clinical photography under consistent lighting and positioning, objective measures (e.g., phototrichogram and hair shaft diameter), and a clear checklist of contraindications, are essential for ethical practice and outcome tracking. Realistic timelines should also be discussed (e.g., detectable improvements with PRP or similar biologically-based injectables often require multiple sessions and may not become evident before 8–12 weeks). To support good clinical practice, we provide (in the Supplementary Material file) a concise sample informed consent form adapted to scalp injection therapies, based on a template available on the Canadian Board of Aesthetic Medicine™ website, as well as a standardized procedure note template that can be adapted to local regulatory and institutional requirements [160]. These documents are provided for practical guidance and are not intended to be binding or exhaustive.

Future research should focus on optimizing injection techniques, developing novel therapeutic agents, and conducting rigorous clinical trials to evaluate the long-term safety and efficacy of scalp injections for hair restoration. Comparative studies evaluating the efficacy of different injection techniques and therapeutic agents are also needed to guide clinical practice. Moreover, the integration of advanced imaging technologies, such as optical coherence tomography (OCT) and confocal microscopy, can enhance the objective assessment of treatment outcomes and facilitate personalized treatment approaches. The identification of biomarkers that predict treatment response can further enhance the precision and efficacy of scalp injection therapies. By addressing these challenges, scalp injections hold the potential to revolutionize hair restoration and provide effective and safe therapeutic options for individuals affected by alopecia.

## 5. Conclusions

In the realm of hair restoration, injectable therapies have undeniably carved a niche as a promising avenue for addressing various forms of alopecia. The current landscape of off-label injectable treatments presents a spectrum of potential benefits, with certain formulations demonstrating encouraging outcomes in terms of stimulating hair regrowth and enhancing scalp health. However, it is imperative to acknowledge that the evidentiary foundation supporting these treatments remains in a state of flux. The paucity of large-scale, long-term clinical trials, which are essential for establishing definitive efficacy and safety profiles across diverse patient demographics, represents a significant gap in our current understanding.

A salient limitation of these topical and injectable therapies is the inherent variability in patient response. This heterogeneity underscores the complex interplay of genetic, hormonal, and environmental factors that influence hair follicle biology. Furthermore, the necessity for repeated injections, often spaced at regular intervals, poses a practical challenge, potentially impacting patient compliance and long-term adherence to treatment regimens. This need for repeated treatments also increases the long-term cost of these therapies.

The economic burden associated with these therapies, coupled with the absence of standardized treatment protocols and dosing guidelines, presents formidable obstacles to broader accessibility and consistent clinical practice. The lack of uniformity in treatment approaches across different clinical settings can lead to inconsistent outcomes and hinder the ability to compare results across studies. This variability also increases the difficulty of determining the true cost effectiveness of each treatment.

To advance the field and solidify the role of injectable therapies in hair restoration, future research endeavors must prioritize the conduct of robust, well-designed clinical trials. These trials should be meticulously designed to compare the efficacy of different injectable treatments in a standardized manner, employing rigorous methodologies and objective endpoints. Such studies should also explore the long-term effects of these treatments, including their durability and potential adverse effects.

Furthermore, research should focus on optimizing injection techniques, dosing schedules, and combination therapies. The development of personalized treatment protocols, tailored to individual patient characteristics and needs, could further enhance therapeutic outcomes. Investigating the synergistic effects of combining injectable therapies with other modalities, such as topical treatments, laser therapy, or oral medications, may also yield significant improvements in hair regrowth and overall patient satisfaction.

Moreover, the elucidation of the precise mechanisms of action of these injectable therapies is crucial for optimizing their efficacy and safety. Understanding the molecular pathways involved in hair follicle stimulation and regeneration can inform the development of more targeted and effective treatments. Research into the identification of biomarkers that predict treatment response can further enhance the precision and personalization of these therapies.

In conclusion, while injectable hair growth products hold considerable promise, continued research and rigorous clinical evaluation are essential to establish their role as a mainstay in hair restoration. The integration of evidence-based practices, standardized protocols, and personalized treatment approaches will be instrumental in maximizing the benefits of these therapies and improving the quality of life for individuals affected by hair loss.

## Figures and Tables

**Table 1:** Overview of representative clinical studies evaluating platelet-rich plasma (PRP) for androgenetic alopecia. All studies declared no adverse events. PRP, platelet-rich plasma; CaCl_2_, calcium chloride; NS, not significant.

Study	Shapiro et al., 2020 [33]	Sasaki et al., 2021 [34]	Gupta et al., 2022 [35]	Nilforoushzadeh et al., 2025 [36]	Gkini et al., 2014 [37]
Kit Used	Regen Blood Cell Therapy kit	Eclipse PRP HC System	Not specified	Kit from Persian Bio-Based Production (PBBP) Company	RegenLab PRP kit
Leukocytes presence	Not stated	Near-absence	Not stated	Not stated	Not stated
Preparation	1× 1500 *g*, 5 min	1× 1500 *g*, 10 min	1500 rpm, 6 min + 2500 rpm, 15 min	160 *g*, 10 min + 400 *g*, 10 min	1500 *g*, 5 min
Activation	Non-activated PRP	Not stated	Activated (CaCl_2_)	Not stated	Activated
Platelet Concentration	Not reported	Batch A: ~27 × 10^6^ platelets/μL Batch B: ~55 × 10^6^ platelets/μL	Not reported	Not reported	1.102 × 10^6^ platelets/μL
Parameters & Dosage	Two 7.6 × 7.6 cm squares; 0.1 mL–0.2 mL/cm^2^	5 mL per hemiscalp (0.05 mL/cm^2^)	3–5 mL injected in a linear fashion about 1 cm apart (0.1 mL/cm^2^)	2 cc of PRP were injected	Nappage technique depth of 1.5–2.5 mm (0.05–0.1 mL/cm^2^)
Injection Depth	Intradermal 3–4 mm (angle 35° to 45°)	Intradermal	Intradermal	Group 1—intradermal injection Group 2—microneedling + topical PRP	Intradermal
Frequency	3 monthly sessions	2 sessions	Group A: PRP every 15 days × 3 months + daily biotin Group B: daily biotin	2 sessions with 1-month interval	3 sessions, 3-week intervals
Outcomes	+20 hairs/cm^2^ vs. placebo +15.7; NS difference	Increase in hair density & follicle diameter; high-dose trend better NS when compared to placebo-control sites	Significant regrowth at 6–12 months (*p* < 0.001) for the Group A (PRP + biotin)	Injected PRP: +62.4% hair count; +58.6% thickness NS differences between groups 1 and 2	Significant increase in hair density (19.29% and 9.19% at 3 and 6 months)

**Table 2:** Listing of market-leading commercial hair boosters. AA, alopecia areata; AGA, androgenetic alopecia; HA, hyaluronic acid; TE, telogen effluvium.

Manufacturer (Location)	Product/Classification	Product Presentation	Active Ingredients	Uses/Applications
Suisselle (Yverdon-les-Bains, Switzerland)	CELLBOOSTER^®^ Hair Class III Medical Device	3 mL vial	Non-cross-linked HA (18 mg), amino-acids, vitamins, copper, zinc	Noncicatricial alopecia, AA, AGA, damaged hair shaft, premature graying, seborrhea, psoriasis
REVITACARE (Saint-Ouen-l’Aumône, France)	HAIRCARE Class III Medical Device	5 mL vial	Non-cross-linked HA (2 mg), amino-acids, B vitamins	Alopecia, scalp health
SELANCARE (Amsterdam, The Netherlands)	DR.CYJ HAIR FILLER Class III Medical Device	1 mL vial	HA (0.7%), phosphate, peptides	Alopecia
Pluryal (Luxembourg)	Pluryal Mesoline Hair Class III Medical Device	5 mL vial	HA, copper peptide, amino-acids, B vitamins, coenzyme Q10, vegetal stem cell booster	AGA, TE
Pluryal (Luxembourg)	Pluryal Hair Density Class III Medical Device	2 mL vial	Polynucleotides (15 mg)	AGA, TE
CROMA Pharma (Leobendorf, Austria)	PhilArt/PolyPhil Hair Class III Medical Device	2 mL pre-filled syringe	Polynucleotides (15 mg)	Female alopecia
INNOAESTHETICS (Barcelona, Spain)	INNO-TDS^®^ HAIR LOSS CONTROL	2.5 mL vial	Fenugreek, capixyl, saw palmetto, azelaic acid, pyridoxine, zinc	Male alopecia
INNOAESTHETICS (Barcelona, Spain)	INNO-TDS^®^ HAIR VITAL	2.5 mL vial	Essential and sulphur amino-acids, trace elements, enzymes, vitamins, peptides	Female alopecia
MCCM Medical Cosmetics (Barcelona, Spain)	MCCM Hair cocktail	10 mL vial	Glutathione, panthenol, biotin, methylsilanol mannuronate	Alopecia
CAREGEN (Gyeonggi-do, Republic of Korea)	Dermaheal HL—Anti Hair Loss	5 mL vial	Biomimetic peptides, vitamins, amino-acids	Alopecia, scalp health

**Table 3:** Listing of the most recent clinical studies for the assessment of hair regrowth of commercially available injectable products. AA, alopecia areata; ADSCs, adipose derived stem cells; AGA, androgenetic alopecia; Pbo, placebo; PRP, platelet-rich plasma; sc, subcutaneous.

ClinicalTrials.gov ID	Phase	Clinical Indications	Intervention/Treatment	Study Details
NCT06444451	II	Severe AA	Amlitelimab	Randomized, double-blind, Pbo-controlled, parallel group, 3-arm, multinational, multicenter, proof-of-concept study to evaluate the efficacy and safety of amlitelimab monotherapy by sc injection. Outcome measure at baseline and weeks 24, 36 and 156.
NCT06340360	IIb	Severe to very severe AA	Rezpegaldesleukin	Randomized, double-blind, parallel group, Pbo-controlled study to evaluate the efficacy and safety of rezpegaldesleukin by sc injection. Outcome measure at baseline and weeks 12, 16, 20, 24, 28, 32, and 36.
NCT06564805	Not applicable	AA	Triamcinolone vs. Candida Albicans Antigen	Comparison between the effectiveness of intralesional triamcinolone and intralesional *Candida albicans* antigen. Outcome measure after 5 months.
NCT06327581	Not applicable	AA	Microneedling with either 1% lactic acid solution or vitamin D3 or triamcinolone acetonide	Comparative study of combined microneedling with either 1% lactic acid solution or vitamin D3 or triamcinolone acetonide or saline 0.9%. Patients in all groups will be subjected to microneedling using a dermapen “Dr. Pen” with adjustable needle length ranging from 1.5 to 2 mm, using the highest speed level (4–5). First, topical anesthetic cream will be applied. Outcome measure after 3 months.
NCT06239207	II	AGA	Exosomes vs. PRP	Group A patients are injected with exosomes, 2 sessions 3 months apart, intradermally at a dose of 0.1 mL/cm^2^ of scalp. Exosomes used are GFC CELL EXO SCALP KIT. PRP, 4 sessions 1 month apart. Group B patients are injected with PRP intradermally into scalp. Outcome measure 6 months after the last session.
NCT06326359	Not applicable	Male AGA	Autologous stromal vascular fraction derived from denovo vs. PRP	Group A will receive 2 sessions of stromal vascular fraction directly after fat harvesting and processing and stromal vascular fraction extraction with 1 month interval. Group B will be injected with 2 sessions of PRP, 3 weeks interval at site of fat harvesting and will be followed 1 month later with fat aspiration from site where PRP was previously injected, followed by 2 sessions of stromal vascular fraction injection in the scalp at 1 month interval. Outcome measures at baseline and at 6 months after treatment.
NCT06018428	IIa	Severe AA	ADX-914	ADX-914 or matching placebo is administered subcutaneously every 2 weeks for 24 weeks, with follow-up for 12 weeks. Outcome measure at week 18, 24 and 40.
NCT05866562	II	Pediatric AA	Dupilumab	Prospective, randomized, double-blind, placebo-controlled clinical trial. Dupilumab 200 mg or 300 mg sc injections every 2 or 4 weeks (weight based). Outcome measure at baseline and week 48 and 96.
NCT06043349	IV	Male AGA	PRP + 5% topical minoxidil vs. 5% topical minoxidil	Effectiveness and safety of PRP and topical 5% minoxidil combination therapy compared with topical 5% minoxidil monotherapy. Outcome measure after 3 months.
NCT06066827	Not applicable	AGA	ADSCs secretome with minoxidil	Effectiveness and safety of ADSCs secretome with minoxidil. Minoxidil 5% solutions for topical use, 1 cc, twice daily, every day for 12 weeks; ADSCs, 2 cc, injected into the scalp on weeks 0, 4, and 8 of the study. Outcome measure at baseline, week 4 after intervention, week 8 after intervention, and week 12 (end of trial).

**Table 4:** List of published consensuses on the optimal clinical use of selected commercial preparations. AGA, androgenetic alopecia; sc, subcutaneous.

Product (Manufacturer)	Volume per Session	Injection Depth	Syringe/Needle Type	Injection Technique	Treatment Protocol	Reference
CELLBOOSTER^®^ Hair (Suisselle)	3 mL	Intradermal	1 mL Luer lock syringes and TSK 33G invisible needles	Micro-papule (at least 50 injection points on the scalp)	Six sessions spaced every two weeks (D0, D14, D21, D28, D42, D56, D70)	[113,139]
HAIRCARE (REVITACARE)	5 mL	Superficial dermis	30G needle	-	6 sessions at intervals of 6 to 10 days	[140,141]
DR.CYJ HAIR FILLER (SELANCARE)	1 mL	Superficial or medium dermis	32G, 30 multi-needle (5 needles of 3 mm), 30° angle	-	4 sessions with 2 weeks interval following 3 sessions spaced 1 month apart	[147]
Pluryal Mesoline Hair (Pluryal)	5 mL	Intradermal	-	Microneedling (roller of 0.5 mm)	6–8 sessions at 10 days interval	[148]
Pluryal Hair Density (Pluryal)	2 mL	Deep dermis	2 × 30G ½ needles	Needle microdroplets	Depends on hair loss type. For AGA: 1 session every 1/2 weeks for a total of 4 sessions followed by 4 sessions performed at 3 to 4-week intervals. To be repeated every year.	[94]
Polynucleotides PN-HPT™ PhilArt hair (CROMA Pharma) 15 mg/2 mL	2 mL	Intradermal	2 × 30G ½, 13 mm	Needle microdroplets (0.2 mL each infiltration)	Initial treatment cycle: one session every 7 or 14 days for a total of 4 sessions. Followed by one session every 21–30 days for a further 4 sessions.	[149,150]
Corticosteroid therapy	2.5–10 mg/mL	Intralesional	-	0.05−0.1 mL per puncture into the dermis or upper part of the sc tissue, with a spacing of 0.5–1 cm between the punctures	Interval of 4 to 6 weeks between sessions. Dilution with saline or glycoside is recommended, and it may or may not be mixed with lidocaine.	[151]

## Data Availability

No new data were created or analyzed in this study.

## Supplementary Materials

The following supporting information can be downloaded at: https://www.mdpi.com/article/10.3390/jcm15051836/s1, Supplementary File S1: This document provides practical templates to support good clinical practice in scalp injection therapies. These templates are provided for guidance only and are not exhaustive nor binding. They should always be adapted to local regulatory and institutional requirements.

## Abbreviations

The following abbreviations are used in this manuscript:

AA	alopecia areata
ADSCs	adipose-derived stem cells
AGA	androgenetic alopecia
AR	androgen receptor
BMSCs	bone marrow-derived stem cells
BTA	Botulinum toxin type A
DHT	dihydrotestosterone
DNA	deoxyribonucleic acid
DPC	dermal papilla cells
EDAR2	ectodysplasin A2 receptor
EGF	epithelial growth factor
FDA	Food and Drug Administration
FPHL	female pattern hair loss
HA	hyaluronic acid
HFSCs	hair follicle stem cells
IGF	insulin-like growth factor
JAK	Janus kinase
MPHL	male pattern hair loss
mRNA	messenger ribonucleic acid
MSCs	mesenchymal stem cells
OCEBM	Oxford Centre for Evidence-Based Medicine
OCT	optical coherence tomography
PDGF	platelet-derived growth factor
PRP	platelet-rich plasma
TE	telogen effluvium
TGF-β1	transforming growth factor beta 1
VEGF	vascular endothelial growth factor
