# Supplementary material for: Advances in Medicalized Hair Loss Solutions: A Review of Current Clinical Practices and Regenerative Medicine-Based Protocols with Focus on Off-Label Injectable Treatments
Source: J Clin Med. 2026 Feb 27;15(5):1836. doi: 10.3390/jcm15051836 (PMC12985832; doi:10.3390/jcm15051836)
Supplement: Supplementary file 1 [file jcm-15-01836-s001.zip › jcm-4158959-supplementary.pdf]

Advances in Medicalized Hair Loss Solutions: A Review of Current Clinical Practices and Regenerative Medicine-Based Protocols with Focus on Off-label Injectable Treatments

Angelica Ferro [1], Mohammad Alkhowailed [2], Alexandre Porcello [1], Marco Cerrano [3], Michèle Chemali [4], Kelly Lourenço [1], Cíntia Marques [1], Wassim Raffoul [5], Lee Ann Applegate [6,7,8] and Alexis E. Laurent [9,10,*]

Supplementary Material

This document provides practical templates to support good clinical practice in scalp injection therapies. These templates are provided for guidance only and are not exhaustive. They should be adapted to local regulatory and institutional requirements.

1. PRP Consent Form

Description of the Procedure

Platelet-Rich Plasma (PRP) therapy is an autologous treatment in which a small volume of the patient's own blood (approximately 20 mL) is collected and processed using centrifugation to separate its components. The platelet-rich fraction is isolated and may be combined with calcium chloride as an activation agent prior to injection.

Platelets contain growth factors and bioactive proteins involved in tissue repair and regeneration. When injected into the scalp, PRP aims to stimulate local biological activity, including angiogenesis, collagen production, and follicular signaling pathways that may support hair growth.

The procedure typically takes 45–60 minutes. A series of 2–3 treatment sessions spaced several weeks apart is commonly recommended. Maintenance sessions may be considered depending on clinical response.

Intended Benefits

Potential benefits may include:

- Improvement in hair density and thickness
- Enhanced scalp tissue quality
- Support of hair follicle activity
- Minimal downtime compared with surgical procedures

Results vary among individuals. PRP is not a substitute for surgical hair restoration when such intervention is clinically indicated.

Evidence and Limitations

PRP has been used for more than two decades in multiple medical specialties for tissue repair applications. In hair restoration, clinical studies suggest potential benefit; however, outcomes may vary due to differences in preparation protocols and patient characteristics.

No guarantee of specific results can be provided.

Contraindications

PRP treatment may not be appropriate for patients with:

- Active infection or systemic illness
- Skin diseases affecting the scalp
- Active malignancy or ongoing chemotherapy
- Severe metabolic or systemic disorders
- Platelet dysfunction syndromes or severe thrombocytopenia
- Chronic liver disease
- Ongoing anticoagulation therapy
- Recent systemic corticosteroid use
- Sepsis

Clinical evaluation is required prior to treatment.

Risks and Potential Complications

As with any injection procedure, possible adverse effects include:

- Pain or discomfort at the injection site
- Bruising, swelling, or bleeding
- Infection (rare)
- Temporary redness or flushing
- Headache, dizziness, or vasovagal reaction
- Minimal or no clinical response

These risks are generally mild and transient but cannot be completely eliminated.

Alternatives

Alternatives to PRP may include:

- No treatment
- Topical therapies (e.g., minoxidil)

- Oral medications (e.g., finasteride, when appropriate)
- Microneedling
- Low-level light therapy
- Botulinum toxin injections
- Hair transplantation surgery

The choice of therapy should be discussed with the treating clinician.

Expected Timeline

Initial changes may become visible after approximately 4–8 weeks. Progressive improvement may occur over 3–6 months. Maintenance treatments may be required to sustain results.

Photography and Documentation

Clinical photographs may be taken for documentation, monitoring of treatment response, and scientific or educational purposes. Patient identity will be protected in all cases unless explicit additional authorization is granted.

Financial Considerations

PRP for hair restoration is an elective procedure and is generally not covered by insurance. Payment policies should be discussed prior to treatment.

Consent Statement

I confirm that:

- I have received and understood information regarding PRP therapy.
- I have had the opportunity to ask questions and received satisfactory answers.
- I understand the potential benefits, limitations, risks, and alternatives.
- I understand that results cannot be guaranteed.
- I voluntarily consent to undergo PRP scalp injection therapy.

Patient Name: ___________________________
Signature: ______________________________
Date: __________________________________

Practitioner Name: ______________________
Signature: ______________________________
Date: __________________________________

Patient Name: ___________________________

Signature: ___________________________

Date: ___________________________

Physician Name: ___________________________

Signature: ___________________________

Date: ___________________________

This consent template is adapted from a publicly available template on the Canadian Board of Aesthetic Medicine™ website. It is provided for guidance only and is not exhaustive.

2. Standardized Procedure Note Template

Patient Information

Name: ___________________________

Date of Birth: ___________________________

Medical Record Number: ___________________________

Date of Procedure: ___________________________

Indication

Diagnosis (e.g., AGA, FPHL, AA): ___________________________

Severity/Stage: ___________________________

Contraindication Checklist

☐ Pregnancy / breastfeeding

☐ Active scalp infection

☐ Bleeding disorders / anticoagulation

☐ Autoimmune or oncologic condition

☐ Other: ___________________________

Procedure Details

Product Used: ___________________________

Lot Number: ___________________________

Total Volume Injected: ___________________________

Injection Technique and Depth: ___________________________

Number of Injection Points: ___________________________

Objective Baseline Measures

☐ Clinical photography completed

☐ Phototrichogram performed

☐ Hair shaft diameter measured

Immediate Tolerance

Pain level (0–10): ______

Immediate adverse events: ___________________________

Post-Procedure Instructions

Instructions provided: ___________________________

Follow-Up Plan

Next appointment: ___________________________

Planned number of sessions: ___________________________

Physician Signature: ___________________________

Date: ___________________________
